# Pemphigus Vulgaris Associated with Rheumatoid Arthritis in a Patient not Taking Penicillamine

**DOI:** 10.5681/joddd.2012.008

**Published:** 2012-03-13

**Authors:** Narges Gholizadeh, Ali Taghavi Zenouz, Hossein Eslami

**Affiliations:** ^1^Assistant Professor, Department of Oral Medicine, Faculty of Dentistry, Tabriz University of Medical Sciences, Tabriz, Iran; ^2^Associate Professor, Department of Oral Medicine, Faculty of Dentistry, Tabriz University of Medical Sciences, Tabriz, Iran

**Keywords:** D-penicillamine, pemphigus vulgaris, rheumatoid arthritis

## Abstract

D-penicillamine is one of the disease-modifying anti-rheumatic drugs (DMARDs). Drug-induced pemphigus is not fre-quently associated with D-penicillamine, and to date, the number of reported cases is about a hundred. Most reports of D-penicillamine-induced pemphigus vulgaris are in patients with rheumatoid arthritis. It has rarely been reported in patients not taking D-penicillamine. We report a case of pemphigus vulgaris in a 48-year-old female patient with rheumatoid arthri-tis, not taking penicillamine.

## Introduction

Pemphigus includes a group of autoimmune, potentially life-threatening diseases that cause blisters and erosions of skin and mucous membranes. The major variants of phemphigus include pemphigus vulgaris (PV), pemphigus foliaceus, paraneoplastic pemphigus (PNPP), and drug-related pemphigus.^1^ The immune system produces antibodies against specific proteins in the skin and mucus membranes. These antibodies break the bonds between skin cells, leading to the formation of a blister. The exact cause is unknown.^2^

Rheumatoid arthritis (RA) is a long-term disease that causes inflammation of the joints and surrounding tissues. It can also affect other organs. RA is an autoimmune disease of unknown cause. RA can occur at any age, but is more common in middle age. Women get RA more often than men. Infection, genes, and hormone changes may be linked to the disease. RA usually affects joints on both sides of the body equally. Wrists, fingers, knees, feet, and ankles are the most commonly affected. The disease often begins slowly, usually with only minor joint pain, stiffness, and fatigue.^3^

The treatment of RA can cause oral manifestations. The long term use of anti-rheumatic agents such as D-penicillamin can cause stomatitis and drug-induced pemphigus.^1^ Drug-induced pemphigus is a well-established variant of pemphigus. A variety of drugs have been implicated in the onset of drug-induced pemphigus. Drugs that induce pemphigus may be categorized into two groups: thiol drugs and non-thiol drugs. Thiol drugs are reported most frequently as the culprits of drug-induced pemphigus. They contain a thiol group (-SH) in their chemical structure. Penicillamine, captopril, and enalapril are the thiol drugs most often associated with drug-induced pemphigus. Nonthiol drugs include sulfur-containing drugs and drugs without sulfur in their structure An active amide group is found in the structure of many nonthiol drugs, which has resulted in the speculation that this structure may be responsible for the induction of disease.^4^

## Case report

A 48-year-old woman was referred to the Department of Oral Medicine, Faculty of Dentistry, Tabriz University of Medical Sciences, for diagnosis and management of a non-painful oral ulceration which had been present for the past six months. The past medical history revealed a diagnosis of RA established five years before, and prednisolone 2.5 mg/d, azathioprine, and methotrexate medications since then.

**Figure 1:**
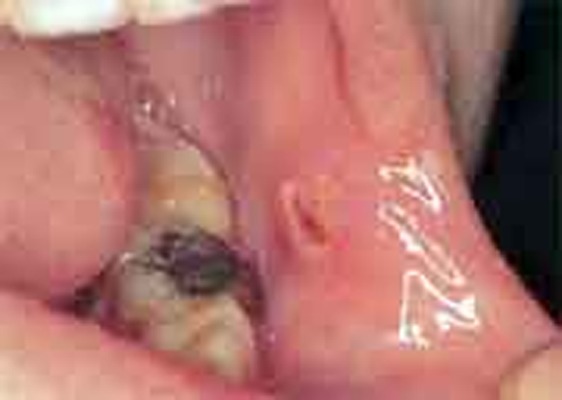
Solitary ulcer on the buccal mucosa of the left side.

Intraorally, along the occlusal line on the buccal mucosa of the left side, there was an ulcerated area measuring approximately 3×3 cm, the margins of which were hyperkeratotic and irregular in outline and the base was covered with a pale yellow-colored slough. The clinical appearance of the intraoral ulcer was more typical of squamous cell carcinoma or a traumatic ulcer because of the lesion’s presence on the line of occlusion (Figure 1).

Under local anesthesia, an elliptical incisional biopsy was taken from the lesion, which included part of the ulcer and normal mucosa. The biopsy specimen was fixed in 10% formalin for conventional histologic examination. Light microscopic examination of sections stained with hematoxylin and eosin showed hyperplastic squamous epithelium and suprabasal bullae with acantholytic cells. There was moderate chronic inflammation in the stroma. Based on these results, a diagnosis of pemphigus was established. Regarding the pathologic diagnosis, the patient was examined for Nikolsky’s sign, which proved negative (Figure 2).


Figure 2. Histopathologic views shows hyperplastic squamous epithelium and suprabasal bullae with acantholytic cells.

**Figure:**
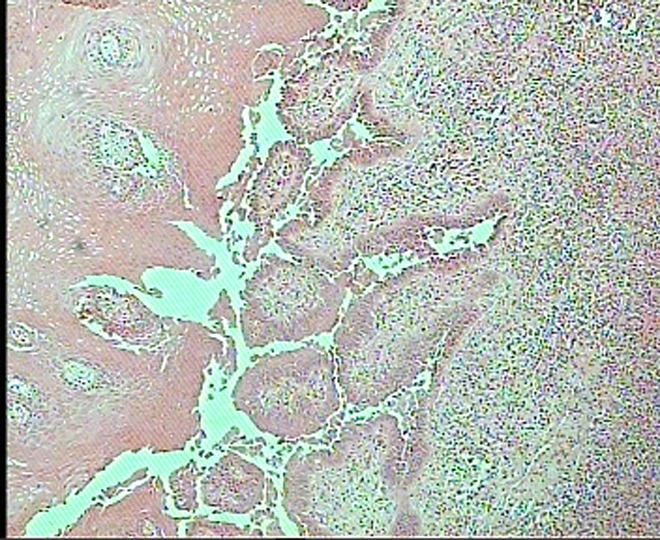

**Figure:**
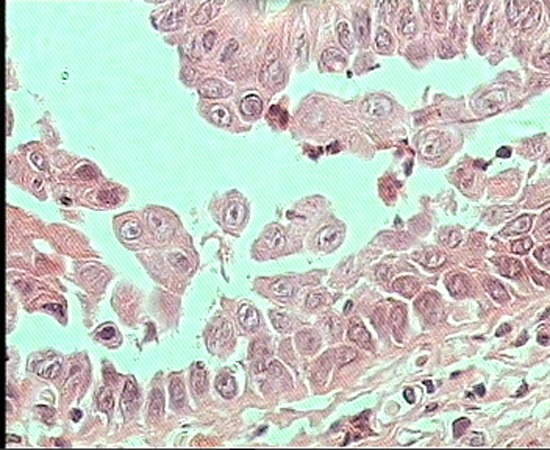

In order to confirm histopathologic diagnosis, the patient was examined for Direct Immunoflurocent (DIF) and Indirect Immunoflurocent (IIF). A biopsy specimen that was obtained from clinically normal appearing peri-lesional mucosa was placed in normal saline solution and was sent for immunofluorescence evaluation for in vivo deposits of IgG, IgM, IgA, IgD, IgE, and complement components of C3 and C4. The patient’s serum was examined by a routine indirect immunofluorescence technique for autoantibodies such as anti-epithelial intercellular and basement membrane antibodies. The findings of DIF and IIF were negative.^1^

The patient in the present case report was scheduled a recall visit after three months, at which the clinical examination did not reveal any changes in the lesion size with no involvement of new locations in the oral mucosa. In addition, no skin involvement was observed and Nikolsky’s sign was negative. Another recall visit was scheduled for the next three months. The patient was asked to return to the clinic before the 3-month follow-up period in case of any oral ulcer or blisters.

In case of the absence of any signs or changes in the variables mentioned above further recall visit will be scheduled for the next 6-month and 1-year periods. The patient has been instructed to pay a recall visit whenever new oral mucous membrane or skill lesions appear, regardless of the visits scheduled for her.

## Discussion

There are several reports on the association of pemphigus with other autoimmune diseases such as RA, thymoma and other malignancies such as lymphoma.^1^

Pemphigus vulgaris might manifest itself in rheumatoid arthritis patients, especially after administration of D-penicillamine.^5 - 11^ The mechanism of drug-induced pemphigus is thought to be the drugs in the thiol group that lead to such lesions due to their potential to induce acantholysis.^12^ In this case, the patient did not receive D-penicillamine for rheumatoid arthritis. The only medications that were prescribed for controlling the disease in the patient were low doses of azathioperin, methotrexate, and prednisolone, all of which are administered in the treatment of rheumatoid arthritis.

The majority of previous reports in the literature have emphasized the concomitant appearance of RA and pemphigus. Wilkinson et al have reported several cases of concomitant occurrence of RA and pemphigus foliaceus.^13^ In cases in which the concomitant occurrence of RA and pemphigus vulgaris have been reported, D-penicillamine or its derivatives have been involved. Jin et al^14^ have reported a case of concomitant RA and pemphigus in a patient receiving bucillamine.

The drugs used in the treatment of RA, such as prednisolone and other immunosuppressive agents, are those used in the treatment of pemphigus. However, the patient was suffering from pemphigus despite the use of such drugs, an observation which might be attributed to the low dose of the drugs used, leading to an atypical and minor form of pemphigus.

Another interesting consideration in the present case report is that five months after the appearance of the oral lesion, the single persistent ulceration was confined to a particular part of the oral mucosa and had not made any progress, which might be attributed to the use of immunosuppressives and corticosteroids administered in the treatment of RA. Regarding the negative results of DIF and IIF in the patient, it should be pointed out that in a small percentage of pemphigus vulgaris patients whose lesions are confined to the oral cavity DIF and IIF tests yield negative results.^1^ In addition, these negative results might be attributed to the above-mentioned medications, taken by the patient. Based on our literature review, this case report is the first case of concomitant occurrence of RA and PV in a patient who had never taken penicillamine. A regular recall program should be in place for such patients to control their condition.

## References

[1] greenberg m, glick m, ship ja (2008). ulcerative, vesicular, and bullous lesions. in: burket lw, lynch ma, eds. burket’s oral medicine: diagnosis and treatment.

[2] habif tp (2009). vesicular and bullous diseases. in: habif tp, ed. clinical dermatology.

[3] huizinga tw, pincus t (2010). in the clinic. rheumatoid arthritis. ann intern med.

[4] wolf r, brenner s (1994). an active amide group in the molecule of drugs that induce pemphigus: a casual or causal relationship. dermatology.

[5] toth gg, jonkman mf (1999). successful treatment of recalcitrant penicillamin-induced pemphigus foliaceus by low-dose intravenous immunoglubulins. br j dermatol.

[6] jan v, callens a, machet l, machet mc, lorette g, vaillant l (1999). d-penicillamin-induced pemphigus, polymyositis and myasthenia. ann dermatol venereal.

[7] bialy-golan a, brenner s (1996). penicillamine-induced bullous dermatoses. j am acad dermatol.

[8] bauer-vinassac d, menkes cj, muller jy, escande jp (1992). hla system and penicillamine induced pemphigus in nine cases of rheumatoid arthritis. scand j rheumatol.

[9] blanken r, doeglas hm, de jong mc, kardaun sh, van leeuwen m (1988). pemphigus-like eruption induced by d-penicillamine and captopril, in the same patient. acta derm venereol.

[10] bahmer fa, bambauer r, stenger d (1985). penicillamin-induced pemphigus foliaceus-like dermatosis. a case with unusual features, successfully treated by plasmapheresis. arch dermatol.

[11] yung cw, hambrick gw jr (1982). d-penicillamin-induced pemphigus syndrome. j am acad dermatol.

[12] ruocco v, sacerdoti g (1991). pemphigus and bullous pemphigoid due to drugs. int j dermatol.

[13] wilkinson sm, smith ag, davis mj, hollowood k, dawes pt (1992). rheumatoid arthritis: an association with pemphigus foliaceus. acta derm venereol.

[14] jin-wuk h, chang-woo l, dae-hyun y (2006). bucillamin-induced pemphigus vulgaris in a patient with rheumatoid arthritis and polymyositis overlap syndrome. j korean med sci.

